# Cultural Appropriateness of an Intervention to Promote Functional Recovery From Hospitalization: Caregiver Views

**DOI:** 10.1093/geroni/igab046.1448

**Published:** 2021-12-17

**Authors:** Rhonda BeLue, Marie Boltz

**Affiliations:** 1 St. Louis University College for Public Health and Social Justice (CPHSJ), St. Louis University, Missouri, United States; 2 Pennsylvania State University, University Park, Pennsylvania, United States

## Abstract

The Fam-FFC model includes caregiver education and care pathway to promote physical function, wellbeing, and cognition. The Ecological Model (EM) provided a framework to assess the cultural appropriateness of the Fam-FFC intervention, through interviews with family caregivers, patients, and nurse champions, and focus groups with staff. Findings are described within the eight dimensions of the EM: 1 ) language: perceptions of the dyads’ comfort level with intervention information; (2) persons: representation of dyads’ ethnic /racial group within the nurse champions’ ethnicity/race; (3) metaphors: use of cultural terms equivalent to those used by participants; (4) content: integration of participants’ values, customs, and traditions in the intervention; (5) concepts: congruence of caregiving concepts with cultural norms; (6) goals: congruence of the intervention goals with participants’ cultural norms and goals; 7) methods: the culturally appropriateness of the delivery of the intervention; and (8) context: alignment of the intervention with the participant’s socio-community context.

